# A Case of Hydrocephalus Internus

**Published:** 1804-05-01

**Authors:** Michael Bartlett

**Affiliations:** Member of the Royal College of Surgeons, London, and Apothecary to the Finsbury Dispensary


### A Case of Hydrocephalus Internus; *communicated by* Michael Bartlett, *Member of the Royal College of Surgeons, London, and Apothecary to the Finsbury Dispensary.*

JAMES WILSON, a boy about eight years of age, became a patient of mine the 19th of June last, for hydrocephalus internus, not with any view towards a cure, but merely to palliate present symptoms; at this time the head was so considerably enlarged, that he could not raise it from the pillow without the assistance of his friends; he had lost the power of speech, and the pain in the head was so excruciating that he would scream for hours at a time, until quite exhausted, when he would sink into a kind of lethargic state, scarcely appearing to breathe.

The account his father gave me of the progress of his complaint previously to my seeing him is as follows. His mother had a very difficult labour with him, (which Mr. Farley, the gentleman who attended her, accounted for by observing that the head was unusually large.) The child grew and was healthy until about sixteen months old, when he was seized with the hooping cough, and that in so violent a degree, that he lost his sight and strength, and became entirely helpless. Mr. F. was requested to see the child, who, after examining the head with some attention, declared it to be hydrocephalus internus. From this time the head continued gradually to enlarge, but the child regained his sight and strength, so as to be enabled to walk with the assistance of his father's hand, or in a go-cart, and that for two hours at the time. His appetite and evacuations were regular; he was lively, and what is very remarkable, notwithstanding the great injury that was done to so delicate an organ as the brain, he retained the sentient faculty, in a manner truly astonishing; for he would reason with his parents (who were of a religious turn of mind) upon the immortality of the soul, and put such questions to them as, to use his father's expression, both astonished and alarmed them. His memory was so retentive, that he would quote passages from scripture which he had heard a year before, and repeat a whole page verbatim, that had been read to him. The remedies made use of (for he had been under a variety of practitioners) were those usually employed upon these occasions, such as blisters, drastic purges, ung. hydr. fort. &c. without any material alteration or benefit to his complaint. A short time previous to my seeing him, the gentleman who attended had ordered his head to be shaved, and some warm stimulating oils to be rubbed on, such as ol. origani, &c. with a view to ease the pain; and what is worthy observation, immediately after one of these rubbings, he lost his speech entirely (which he had not done before) and never regained it after. From the time I first saw him until his death, which happened the 15th of October following, he remained much in the same state, sensible but speechless, vision imperfect but not obliterated, as he could distinguish objects of moderate magnitude.

*Phenomena that appeared on Dissection.*

The skull very thin and transparent, not being thicker than a shilling in some parts. Sutures closely united. The vessels upon the dura mater rather turgid, but that membrane did not adhere to the cranium so firm as is usual at that age. Longitudinal sinus natural. Upon raising the dura mater, the convoluted appearance of the brain was lost for a smoother surface, and the vessels of the pia mater lying upon that surface. The cerebrum had a very flaccid feel, and the undulating motion of the water was evident through it; it was necessary before cutting through the falciform process of the dura mater to support the depending part of the brain, upon attempting to remove the upper part of the right hemisphere. Although the incision was made two inches above the corpus callosum obliquely upwards and outwards, it opened the right lateral ventricle, and part of the water escaped. From the under side of the corpus callosum, about two inches above the upper side of the fornix, the two lamina of the septum lucidum were distinctly seen, and the handle of the knife could with ease be passed between them. The membrane was quite transparent; the communication of the two lateral ventricals was at the posterior part. The water of a light straw colour, and about *thirty-two ounces* in quantity. No appearance of inflammation upon the membrane lining the ventricles; the communication to the third ventricle considerably enlarged, as was that cavity. The internal surface of the cerebellum natural, but at the posterior part a pellucid membrane appeared, under which was contained about eight ounces of water of a much darker colour than that in the lateral ventricles; this was contained in a cyst immediately under the tentorium, and had no outlet or

 comcommunication with the fourth ventricle whatever.* The optic nerves were smaller, softer, and of a browner colour than natural. The other nerves were natural.

*On the Nature and Cure of the Complaint.*

From the history of this case it does appear, that the disease existed at birth, a circumstance which I conceive generally, if not *always*, to take place, as hydrocephalus is decidedly, in my opinion, a modification of scrophula, proceeding from stamina originally debilitated and relaxed. Admitting this to be the case, ought we not to pay a more strict regard to the original state of the constitution, and by prescribing such remedies (in addition to topical ones) as are most likely to restore the vigour and tone of the constitution, strike at the root of the disease: such as country air, sea bathing, nourishing diet, &c.? Ferrum præp. is a most excellent medicine for children of weak and relaxed habits, given in doses of from three to six grains two or three times a day. Superficial symptoms are too much attended to in practice, while the more important, the actual and existing state of the constitution, is too often neglected; the strength or the weakness of the patient is a grand point to be attended to in the treatment of every disorder, however different in their symptoms and appearances. To quote the words of a monthly medical reporter: "Notwithstanding a considerable diversity in their shapes and complexion, there exist in strictness of fact, only *two families* of disease. The one, the offspring of debility; the other owing its immediate origin to a state of undue mental or physical excitement. This simple doctrine, first started and promulgated by the celebrated Brown, throws upon the obscure region of medical science a beam of light, which upon almost every possible occurrence or supposable emergency may securely guide the judgment and conduct of an experienced, careful, and discriminating practitioner."†

*St. John's Square, March* 23, 1804.

---

* This cyst is in the possession of my friend, Mr. Taunton, of Paternoster Row, a gentleman to whom I have been on various occasions obliged for important information, and who by his persevering zeal in anatomical research, promises to be no small contributor to the promotion of professional science.

† See Dr. Reid's Account of Diseases, Monthly Magazine for July, 1803.

